# Use of PET Imaging to Assess the Efficacy of Thiethylperazine to Stimulate Cerebral MRP1 Transport Activity in Wild-Type and APP/PS1-21 Mice

**DOI:** 10.3390/ijms23126514

**Published:** 2022-06-10

**Authors:** Michael Wölfl-Duchek, Severin Mairinger, Irene Hernández-Lozano, Thomas Filip, Viktoria Zoufal, Mathilde Löbsch, Johann Stanek, Claudia Kuntner, Thomas Wanek, Martin Bauer, Jens Pahnke, Oliver Langer

**Affiliations:** 1 Department of Clinical Pharmacology, Medical University of Vienna, 1090 Vienna, Austria; michael.woelfl-duchek@meduniwien.ac.at (M.W.-D.); severin.mairinger@meduniwien.ac.at (S.M.); irene.hernandezlozano@meduniwien.ac.at (I.H.-L.); martin.m.bauer@meduniwien.ac.at (M.B.); 2 Department of Biomedical Imaging und Image-Guided Therapy, Division of Nuclear Medicine, Medical University of Vienna, 1090 Vienna, Austria; johann.stanek@meduniwien.ac.at (J.S.); claudia.kuntner@meduniwien.ac.at (C.K.); thomas.wanek@meduniwien.ac.at (T.W.); 3 Preclinical Molecular Imaging, AIT Austrian Institute of Technology GmbH, 2444 Seibersdorf, Austria; thomas.filip@meduniwien.ac.at (T.F.); viktoria.zoufal@chello.at (V.Z.); mathilde.loebsch@meduniwien.ac.at (M.L.); 4 Core Facility Laboratory Animal Breeding and Husbandry, Medical University of Vienna, 1090 Vienna, Austria; 5 Department of Neuro-/Pathology, Oslo University Hospital (OUS), University of Oslo (UiO), 0424 Oslo, Norway; jens.pahnke@medisin.uio.no; 6 LIED, University of Lübeck, 23562 Lübeck, Germany; 7 Department of Pharmacology, Faculty of Medicine, University of Latvia, 1586 Rīga, Latvia; 8 Department of Neurobiology, The George S. Wise Faculty of Life Sciences, Tel Aviv University, Tel Aviv 69978, Israel

**Keywords:** MRP1 stimulation, PET, brain, Alzheimer’s disease, amyloid-beta, thiethylperazine, 6-bromo-7-[^11^C]methylpurine

## Abstract

Multidrug resistance-associated protein 1 (MRP1, encoded by the *ABCC1* gene) may contribute to the clearance of amyloid-beta (Aβ) peptides from the brain into the blood and stimulation of MRP1 transport activity may be a therapeutic approach to enhance brain Aβ clearance. In this study, we assessed the effect of thiethylperazine, an antiemetic drug which was shown to stimulate MRP1 activity in vitro and to decrease Aβ load in a rapid β-amyloidosis mouse model (APP/PS1-21), on MRP1 transport activity by means of positron emission tomography (PET) imaging with the MRP1 tracer 6-bromo-7-[^11^C]methylpurine. Groups of wild-type, APP/PS1-21 and *Abcc1^(−/−)^* mice underwent PET scans before and after a 5-day oral treatment period with thiethylperazine (15 mg/kg, once daily). The elimination rate constant of radioactivity (*k*_elim_) was calculated from time–activity curves in the brain and the lungs as a measure of tissue MRP1 activity. Treatment with thiethylperazine had no significant effect on MRP1 activity in the brain and the lungs of wild-type and APP/PS1-21 mice. This may either be related to a lack of an MRP1-stimulating effect of thiethylperazine in vivo or to other factors, such as substrate-dependent MRP1 stimulation, insufficient target tissue exposure to thiethylperazine or limited sensitivity of the PET tracer to measure MRP1 stimulation.

## 1. Introduction

Multidrug resistance-associated protein 1 (MRP1, encoded in humans by the *ABCC1* gene and in rodents by the *Abcc1* gene) is an adenosine triphosphate (ATP)-binding cassette (ABC) transporter with an ubiquitous tissue distribution [1,2]. It uses the energy of ATP to transport its substrates across cellular membranes. MRP1 can transport certain anticancer drugs (e.g., etoposide, vincristine and doxorubicin) and has been implicated in tumor multidrug resistance. Moreover, MRP1 transports several endogenous signaling molecules (e.g., cysteinyl leukotriene C4) and plays a role in cellular response to oxidative stress and inflammation [2]. It has also been suggested that MRP1 contributes, along with P-glycoprotein (P-gp, encoded in humans by the *ABCB1* gene and in rodents by the *Abcb1a* and *Abcb1b* genes), to the clearance of amyloid-beta (Aβ) peptides across the blood-brain barrier (BBB) and blood-cerebrospinal fluid barrier (BCSFB) into the blood [3]. In a rapid β-amyloidosis mouse model (APP/PS1-21), knockout of the *Abcc1* gene led to 12–14-fold increases in brain Aβ levels as compared with MRP1-competent control mice [3]. Moreover, treatment of APP/PS1-21 mice with thiethylperazine, an antiemetic and antidopaminergic drug approved by the Food and Drug Administration and in vitro stimulator of MRP1 transport activity [4], markedly reduced Aβ load in APP/PS1-21 mice expressing MRP1 but not in such mice lacking MRP1 [3]. It has been hypothesized that the brain Aβ-lowering effect of thiethylperazine was caused by a stimulation of cerebral MRP1 transport activity leading to increased elimination of Aβ from the brain. A clinical study has been conducted in patients with early to mild Alzheimer’s disease (AD) to assess the safety and efficacy of thiethylperazine to increase the efflux of Aβ peptides into the blood (DrainAD, ClinicalTrials.gov identifier: NCT03417986). However, to prove the proposed mechanism of action of thiethylperazine an in vivo measure of cerebral MRP1 activity would be required, which was not included in the clinical study.

Positron emission tomography (PET) imaging with 6-bromo-7-[^11^C]methylpurine ([^11^C]BMP) has been used to measure the activity of MRP1 in the brain and lungs of mice and rats [5,6,7,8,9,10,11]. [^11^C]BMP itself is not transported by MRP1 but is in vivo rapidly and quantitatively converted by cytosolic glutathione-*S*-transferases into the corresponding glutathione conjugate S-(6-(7-[^11^C]methylpurinyl)) glutathione ([^11^C]MPG), which is a substrate of MRP1 [9]. PET scans after intravenous (i.v.) injection of [^11^C]BMP revealed a several-fold reduction in the elimination rate constant of radioactivity (*k*_elim_) from the brain and lungs in *Abcc1* knockout mice (*Abcc1^(−/−)^*) relative to wild-type mice, which suggested that MRP1 plays a crucial role in the tissue elimination of the radiolabeled glutathione conjugate of [^11^C]BMP [5,6,8,9]. HPLC analysis revealed that the radiolabeled glutathione conjugate [^11^C]MPG was the only chemical species present in mouse brain at 15 and 60 min after i.v. injection of [^11^C]BMP [9].

The aim of the present study was to use [^11^C]BMP PET to assess the effect of oral treatment with thiethylperazine on MRP1 transport activity in the brain and other tissues in wild-type and APP/PS1-21 mice. As a negative control, a group of *Abcc1^(−/−)^* mice was also included.

## 2. Results

**Figure 1:**
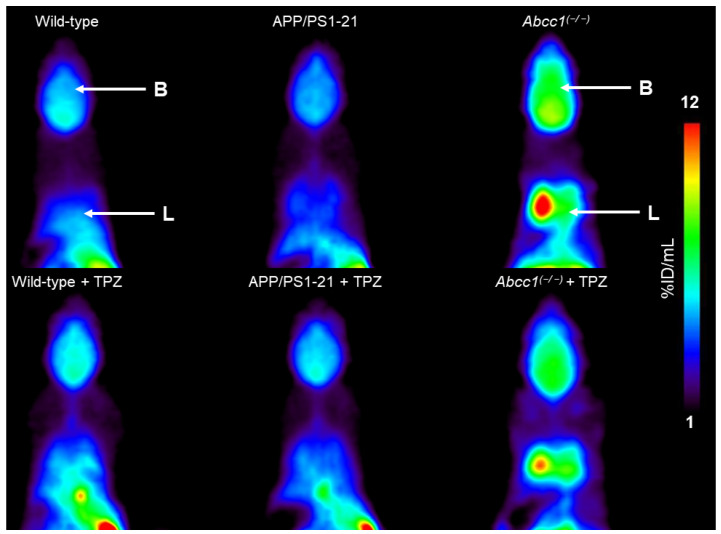
Representative coronal PET summation images (0–90 min) of one wild-type, APP/PS1-21 and *Abcc1^(−/−)^* mouse at baseline and following 5-day oral treatment with thiethylperazine (TPZ, 15 mg/kg, once daily). Anatomical regions are labeled with arrows (B, brain; L, lungs). Radioactivity concentration is expressed as percent of the injected dose per milliliter tissue (%ID/mL). All images are scaled to the same intensity (1–12 %ID/mL).

Groups of wild-type, APP/PS1-21 and *Abcc1^(−/−)^* mice underwent [^11^C]BMP PET scans before and after a 5-day oral treatment period with thiethylperazine (15 mg/kg, once daily). An overview of the animals examined in this study is given in Table 1. Thiethylperazine treatment was well tolerated in all animals. Two out of seven wild-type animals did not complete the second PET scan as they died shortly after radiotracer injection. Coronal PET summation images obtained after injection of [^11^C]BMP are shown in Figure 1 for one representative animal of each group, both before and after thiethylperazine treatment.

**Figure 2:**
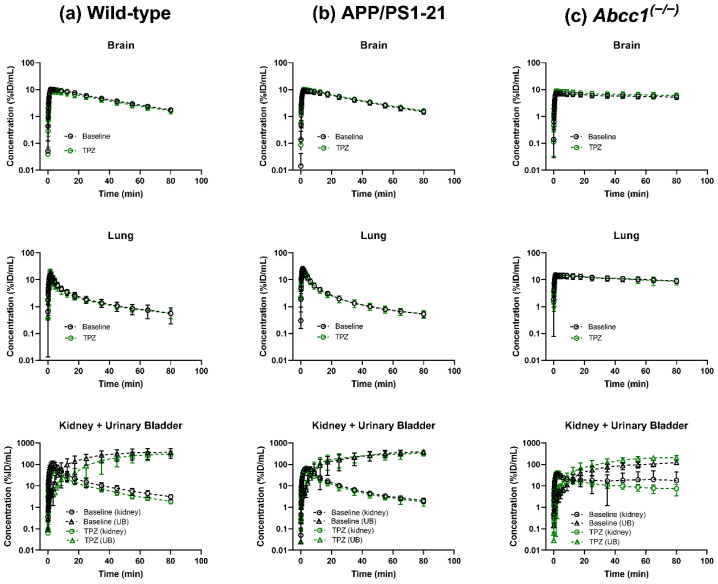
Time-activity curves (mean ± SD) measured after i.v. injection of [^11^C]BMP in the brain, the right lung, and the left kidney and urinary bladder (UB) of wild-type mice (**a**), APP/PS1-21 mice (**b**) and *Abcc1^(−/−)^* mice (**c**) at baseline and following 5-day oral treatment with thiethylperazine (TPZ, 15 mg/kg, once daily).

As described in previous studies [5,6,11], *Abcc1^(−/−)^* mice had higher radioactivity concentrations in the brain and the lungs than wild-type and APP/PS1-21 mice, which corroborated the crucial role of MRP1 in the tissue elimination of [^11^C]BMP-derived radioactivity. In all three mouse groups, no major differences in radioactivity distribution were evident before and after thiethylperazine treatment (Figure 1). Time-activity curves (TACs) for brain, lungs, and kidneys and urinary bladder (i.e., the main excretory organs of [^11^C]BMP-derived radioactivity) are shown in Figure 2. In the brain, lungs and kidneys the elimination of radioactivity was slower in *Abcc1^(−/−)^* mice than in wild-type mice.

**Figure 3:**
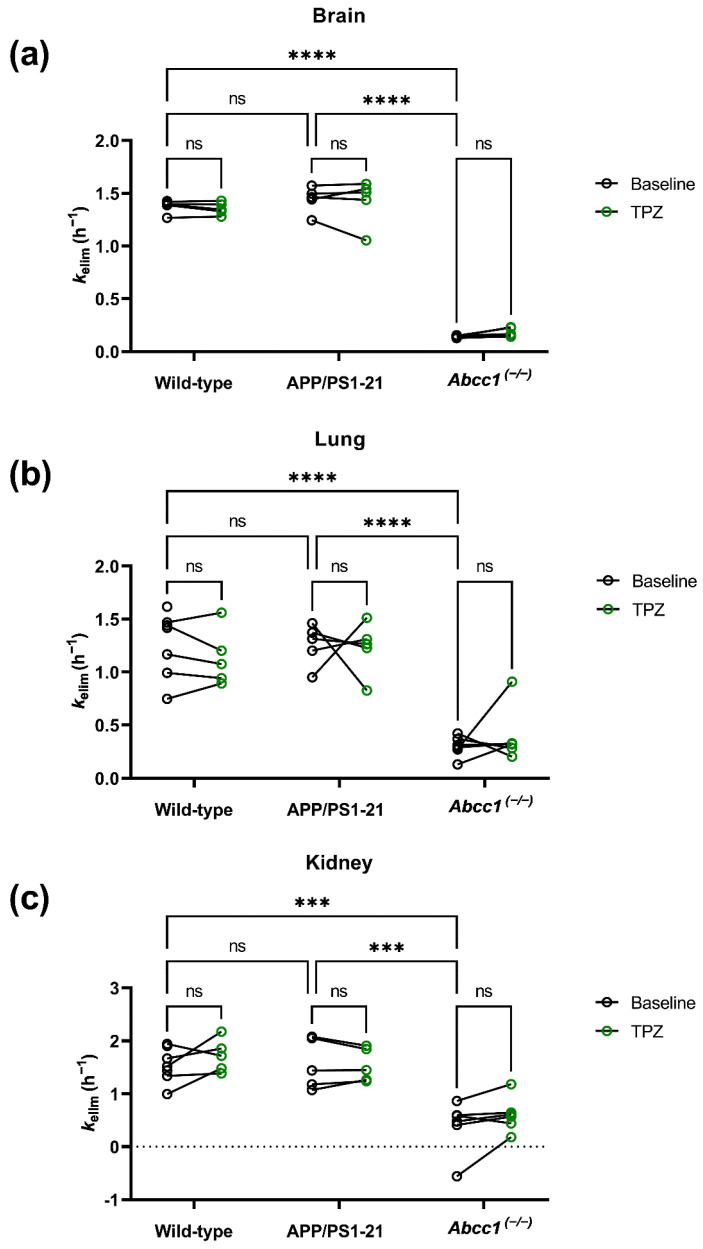
Elimination rate constants of radioactivity (*k*_elim_) from the brain (**a**), the right lung (**b**) and the left kidney (**c**) in wild-type, APP/PS1-21 and *Abcc1^(−/−)^* mice at baseline and following 5-day oral treatment with thiethylperazine (TPZ, 15 mg/kg, once daily). ns, not significant, *** *p* ≤ 0.001, **** *p* ≤ 0.0001, two-way ANOVA followed by a Šidák’s multiple comparisons test.

As an outcome parameter of tissue MRP1 activity, the elimination rate constant of radioactivity (*k*_elim_) was calculated, corresponding to the slope of the late linear part of the log-transformed TACs (Table 2, Figure 3). As reported in previous studies [5,6,11], *k*_elim_ values in brain, lungs and kidneys were significantly lower in *Abcc1^(−/−)^* mice than in wild-type and APP/PS1-21 mice (Figure 3). However, no significant differences were found in *k*_elim_ values between wild-type and APP/PS1-21 mice for all three studied organs. Thiethylperazine treatment led to no significant changes in *k*_elim_ values as compared to baseline scans in all three animal groups, for all three studied organs (Figure 3).

We sampled blood at the end of each PET scan and measured radioactivity in blood and plasma samples in a gamma counter (Table 3). Blood and plasma radioactivity concentrations and plasma-blood concentration ratios were not significantly different before and after thiethylperazine treatment in all three animal groups. Blood concentrations were significantly higher (*p* < 0.0001) and plasma-blood ratios were significantly lower (*p* < 0.0001) in *Abcc1^(−/−)^* mice than in wild-type and APP/PS1-21 mice, while plasma concentrations were not different. As discussed earlier, this phenomenon may be related to an influence of MRP1 on the accumulation of [^11^C]BMP-derived radioactivity in blood cells [5].

## 3. Discussion

In this study, we investigated with [^11^C]BMP PET the ability of thiethylperazine to stimulate MRP1 transport activity in the brains of wild-type and APP/PS1-21 mice. The main finding of our study was the apparent lack of an MRP1-stimulating effect of thiethylperazine, which questions the efficacy of this drug as a MRP1 stimulator in AD patients.

There is a high and unmet medical need for disease-modifying drugs for the treatment of AD. Building on the amyloid cascade hypothesis of AD [12], the most investigated therapeutic target has so far been Aβ accumulation in the brain [13]. To this end, either drugs which inhibit the production of Aβ peptides (e.g., beta-site amyloid precursor protein cleaving enzyme 1 (BACE1) inhibitors) or drugs that promote clearance of Aβ from the brain (anti-Aβ immunotherapies) have been investigated. Unfortunately, these drugs have hardly provided any clinical benefit [13], which emphasizes the need for alternative treatment approaches. Several lines of evidence suggest that ABC transporters such as P-gp and MRP1 may critically contribute to the elimination of neurotoxic Aβ peptides from the brain into the blood and that these transport mechanisms may be potentially therapeutically targeted [14]. Numerous signaling pathways that regulate the activity of ABC transporters have been described [15] and pharmacological approaches to target these pathways have been assessed. For instance, activation of the nuclear receptor pregnane X receptor (PXR) with 5-pregnen-3β-ol-20-one-16α-carbonitrile was shown to enhance P-gp transport activity in β-amyloidosis mouse models, measured either in isolated brain capillaries or in vivo with PET, with a concomitant decrease in brain Aβ load [16,17]. In contrast to the firmly established role of P-gp in brain Aβ clearance [16,18,19,20], much less is known with respect to the role of MRP1 in brain Aβ clearance, other than the observation that knockout of the *Abcc1* gene in APP/PS1-21 mice led to a higher build-up of Aβ load in the brain than knockout of the *Abcb1a/b* genes [3]. MRP1 is abundantly expressed in the basolateral membrane of choroid plexus epithelial cells forming the BCSFB as well as in glial cells, but it remains somewhat controversial whether MRP1 is expressed only in the abluminal or both in the abluminal and luminal membranes of brain capillary endothelial cells [21,22]. Similar to the induction of P-gp activity, induction of cerebral MRP1 activity may be potentially pursued as a strategy to enhance Aβ clearance from the brain. In contrast to P-gp, less information is available regarding signaling pathways that regulate MRP1 activity [23]. In this context, the choice of an inducing agent also has to consider its potential for clinical applicability, for which the re-purposing of approved drugs appears attractive.

Thiethylperazine is an old-generation antiemetic drug, which can be administered both orally and by intramuscular injections at a clinical dose of 10 mg (see: https://www.drugs.com/pro/torecan.html (accessed on 8 June 2022)). Thiethylperazine inhibits dopamine D_2_ receptors in the medullary chemoreceptor trigger zone, thereby decreasing stimulation of the vomiting center in the brain. It exhibits central nervous system (CNS) side effects (i.e., extrapyramidal symptoms) caused by inhibition of dopamine D_2_ receptors in the basal ganglia, which suggests BBB permeability of the drug. One study has reported that thiethylperazine (5–15 µmol/L) can stimulate in vitro the efflux of the MRP1 substrate 2′,7′-bis-(3-carboxypropyl)-5(6)-carboxy-fluorescein (BCPCF) from human erythrocytes, and that this effect was abolished in presence of the MRP1 inhibitors orthovanadate or benzbromarone [4]. In another study, thiethylperazine at a concentration of approximately 2 µmol/L was shown to enhance in vitro the stimulation of human MRP1 ATPase activity by the MRP1 substrate *N*-ethylmaleimide glutathione (NEM-GS), while this effect was not seen in absence of NEM-GS. [3] This suggested that thiethylperazine is a stimulator of MRP1 transport activity but not a substrate of MRP1. The mechanism of the MRP1 transport-stimulating effect of thiethylperazine has not been elucidated yet, but may be similar to the known phenomenon of xenobiotic-stimulated glutathione transport by MRP1 [1,2]. Krohn et al. [3] reported that treatment of APP/PS1-21 mice for 30 days with thiethylperazine twice daily at a dose of 3 mg/kg (intramuscularly) starting at the age of 45 days significantly decreased brain Aβ42 levels in APP/PS1-21 mice but not in APP/PS1-21 x *Abcc1^(−/−)^* mice. In a post-onset treatment paradigm, APP/PS1-21 mice received once daily oral treatment with a dose of 15 mg/kg from an age of 75 days to 100 days, which resulted in a significant decrease in buffer-soluble Aβ and plaque burden [3]. Based on these encouraging non-clinical in vitro and in vivo results, a clinical study has been conducted, in which AD patients were treated for 54 days with a daily oral dose of 26 mg of thiethylperazine (NCT03417986), for which no results have been reported yet.

To elucidate whether thiethylperazine can stimulate cerebral MRP1 transport activity in vivo in mice, we treated in the present study wild-type and APP/PS1-21 mice with the same oral dose as employed by Krohn et al. [3] and measured cerebral MRP1 transport activity with [^11^C]BMP PET at baseline and after the end of treatment. In contrast to the study by Krohn et al. [3], we focused on the MRP1-stimulating and not on the Aβ-lowering effect of thiethylperazine, and therefore, employed only a short-term oral thiethylperazine treatment schedule (5 days), which we considered sufficient to cause MRP1 stimulation, but which was most likely not sufficient to observe reductions in brain Aβ load. Moreover, in contrast to the study by Krohn et al. [3] in which mice with a FVB/N genetic background were used, we used mice with a C57BL/6J genetic background. The glutathione conjugate of [^11^C]BMP ([^11^C]MPG) was shown to be a substrate of mouse, rat and human MRP1 [10,11,24,25] and its brain elimination was by several-fold reduced in *Abcc1^(−/−)^* mice as compared with wild-type mice [5,6,9]. These results were confirmed in the present study, showing significantly lower *k*_elim_ values in brains of *Abcc1^(−/−)^* mice as compared with wild-type and APP/PS1-21 mice (Figure 3). In agreement with the results of a previous study, in which significant differences in cerebral MRP1 activity between APP/PS1-21 mice and wild-type mice could be only detected after partial inhibition of MRP1 with MK571 [6], brain *k*_elim_ values were in our study not significantly different between APP/PS1-21 mice and wild-type mice (Figure 3). Okamura et al. have shown that other than MRP1, organic anion transporter 3 (OAT3, encoded by the *Slc22a8* gene) and multidrug resistance-associated protein 4 (MRP4, encoded by the *Abcc4* gene) contributed to the elimination of [^11^C]BMP-derived radioactivity from the mouse brain [7]. It has been proposed that MRP1 mediates the efflux of [^11^C]MPG from brain parenchymal cells (i.e., astrocytes, neurons), while OAT3 and MRP4 mediate its transport across the abluminal and luminal membranes of brain capillary endothelial cells, respectively. As the brain elimination rate of radioactivity after intracerebral injection of the glutathione conjugate [^11^C]MPG (which by-passed MRP1-mediated efflux from brain parenchymal cells) was similar to that after i.v. injection of [^11^C]BMP, it has been questioned whether MRP1 activity is the rate-determining step in the brain elimination of [^11^C]BMP-derived radioactivity [7,26]. This suggested a limited sensitivity of [^11^C]BMP to measure moderate changes in cerebral MRP1 activity in mice. On the other hand, experiments in heterozygous *Abcc1* knockout mice (*Abcc1^(+/−)^*), which presumably have a 50% reduction in cerebral MRP1 expression as compared with wild-type mice, and experiments after treatment of wild-type mice with MK571, showed significant decreases in brain *k*_elim_ values, indicating that [^11^C]BMP PET is sensitive to detect a decrease in MRP1 expression as well as MRP1 inhibition in vivo [5]. However, the ability of [^11^C]BMP PET to measure pharmacological MRP1 induction in the brain has not yet been investigated.

In the present study, oral treatment with thiethylperazine was found to exert no significant effect on brain *k*_elim_ values in both wild-type and APP/PS1-21 mice (Figure 3). This may indicate that thiethylperazine was not able to stimulate MRP1 transport activity in the mouse brain at the investigated dose and that the Aβ-lowering effect of thiethylperazine reported by Krohn et al. [3] in APP/PS1-21 mice was due to other effects than enhanced brain Aβ efflux by MRP1 stimulation. On the other hand, as MRP1 is known to contain at least three different substrate/modulator binding sites [27], it cannot be excluded that the MRP1-stimulating effect of thiethylperazine is substrate-dependent, so that transport of BCPCF and NEM-GS may be stimulated [3,4] while transport of [^11^C]MPG may not be stimulated. However, this possibility appears unlikely as glutathione conjugates appear to share the same substrate binding site on MRP1 [27] and both NEM-GS and MPG are glutathione conjugates. To investigate this further, in vitro transport experiments would be required to compare the ability of thiethylperazine to stimulate MRP1 transport of different substrates including MPG as well as Aβ peptides. Another possible reason for the observed in vitro-in vivo discrepancy with respect to the MRP1-stimulating effect of thiethylperazine may be related to the inability of thiethylperazine to achieve sufficiently high concentrations at its effect site in the mouse brain. No data on clinical pharmacokinetics and BBB permeability of thiethylperazine are available from the literature. CNS adverse effects (extrapyramidal symptoms) observed in the clinic suggest BBB permeability, but unbound brain concentrations of thiethylperazine after oral treatment of mice with the 15 mg/kg dose are unknown. One study reported a blood concentration of 3.2 ng/mL (5 nmol/L) of thiethylperazine at 3 h after administration of a single oral dose of 10 mg to one healthy male volunteer [28], which is below the concentration range in which Wesolowska et al. [4] and Krohn et al. [3] observed MRP1 stimulation in their in vitro assays (2–15 µmol/L). Even if there are no major species differences in the pharmacokinetics of thiethylperazine, the 15 mg/kg oral dose administered to mice (approximately 35-times higher than the human oral dose of 26 mg employed in the clinical study), may not provide the plasma concentration range in which in vitro MRP1 stimulation was observed (2–15 µmol/L) [3,4].

Other than the brain, MRP1 is also abundantly expressed in lung epithelial cells [29] and the lung elimination of [^11^C]BMP-derived radioactivity was shown to be markedly reduced in mice and rats when either the *Abcc1* gene was knocked out or MRP1 was pharmacologically inhibited with MK571, both after i.v. (mice, rats) and intratracheal radiotracer administration (rats) [5,8,10,11]. We therefore also assessed in the present study the effect of thiethylperazine on the elimination of [^11^C]BMP-derived radioactivity from the lungs. Similar to the brain, no effect of thiethylperazine treatment was observed (Figure 3), which may indicate that restricted BBB permeability was not the reason for the lack of an in vivo MRP1-stimulating effect of thiethylperazine.

Previous studies have shown that [^11^C]BMP-derived radioactivity was mainly excreted into the urine in mice and experiments in *Abcc4^(−/−)^* mice indicated that MRP4, which is localized in the brush border membrane of kidney proximal tubule cells, may contribute to the tubular secretion of [^11^C]BMP-derived radioactivity [5]. We thus assessed the effect of thiethylperazine on *k*_elim_ values in the kidneys, which also remained unchanged (Figure 3). In addition, TACs derived from the urinary bladder were in all animal groups similar before and after thiethylperazine treatment (Figure 2), which suggested that the amount of radioactivity excreted into the urine remained unchanged.

Limitations of our study include the lack of in vitro experiments to assess the ability of thiethylperazine to stimulate transport of MPG by mouse and human MRP1 and the lack of thiethylperazine concentration measurements in mouse brain and plasma.

## 4. Materials and Methods

### 4.1. Chemicals

Unless otherwise stated, all chemicals were purchased from Sigma-Aldrich Chemie (Schnelldorf, Germany) or Merck (Darmstadt, Germany). Torecan^®^ ampoules for intramuscular administration containing an aqueous solution of thiethylperazine maleate (20 mg/2 mL) were obtained from Novartis AG (Basel, Switzerland) and diluted with sterile water (1:1) to a final concentration of 2.5 mg/mL for oral administration to mice.

### 4.2. Radiotracer Synthesis

6-Bromo-7-[^11^C]methylpurine ([^11^C]BMP) was synthesized as previously described [5]. [^11^C]BMP was formulated in 0.9% (*w/v*) aqueous saline for i.v. injection into mice. Radiochemical purity of [^11^C]BMP was greater than 98% and molar activity at the end of synthesis was >50 GBq/µmol.

### 4.3. Animals

Female wild-type mice, female transgenic mice expressing mutated human amyloid precursor protein (APP) and presenilin 1 (PS1) under control of the Thy1-promoter (APP_KM670/671NL_, PS_L166P_) (referred to as APP/PS1-21 mice) [30] and female and male *Abcc1^(−/−)^* mice, all with a C57BL/6J genetic background, were generated at the KPM Radium Hospital (Oslo, Norway). At the start of the experiment, mean age was 158 ± 7 days (range: 149–167 days) for wild-type mice, 158 ± 0 days (range: 158–159 days) for APP/PS1-21 mice and 135 ± 37 days (range: 84–161 days) for *Abcc1^(−/−)^* mice. Animals were housed in type III IVC cages under controlled environmental conditions (22 ± 3 °C, 40–70% humidity, 12-hour light/dark cycle) and had free access to standard laboratory rodent diet (ssniff R/M-H, ssniff Spezialdiäten GmbH, Soest, Germany) and water. An acclimatization period of at least one week was allowed before the animals were used in the experiments. The study was approved by the national authorities (Amt der Niederösterreichischen Landesregierung, approval number: LF1-TVG-48/006-2015, date of approval: 30 March 2015) and study procedures were in accordance with the European Communities Council Directive of 22 September 2010 (2010/63/EU). The animal experimental data reported in this study are in compliance with the ARRIVE (Animal Research: Reporting in Vivo Experiments) guidelines.

### 4.4. Experimental Design

Three groups of mice (wild-type, APP/PS-21 and *Abcc1^(−/−)^*) underwent a baseline PET scan after intravenous (i.v.) administration of [^11^C]BMP. Seven days after the baseline scan, animals underwent a second PET scan after i.v. administration of [^11^C]BMP. Starting two days after the baseline PET scan and for five consecutive days until the day before the second PET scan, animals underwent once daily treatment (at 4 pm) with thiethylperazine solution by oral gavage (6 mL/kg body weight, corresponding to a dose of 15 mg/kg of mean body weight of all animals at the beginning of the treatment period). The dose of thiethylperazine was chosen based on a previous work [3].

### 4.5. PET Imaging

PET imaging was conducted under isoflurane/air (2.5–3.5%) anesthesia on a microPET Focus220 scanner (Siemens Medical Solutions, Knoxville, TN, USA). Animals were warmed throughout the experiment while constantly monitoring body temperature and respiratory rate. [^11^C]BMP was administered in a volume of 0.1 mL as an i.v. bolus (see Table 1 for injected activities). A dynamic 90-minute PET scan was initiated at the start of radiotracer injection. List mode data were acquired with a timing window of 6 ns and an energy window of 250–750 keV. At the end of each PET scan, a blood sample was collected from the retro-bulbar plexus. Blood was centrifuged to obtain plasma and radioactivity in blood and plasma samples was measured in a gamma counter (Wizard 1470; Perkin-Elmer, Waltham, MA, USA). Following the second PET scan, animals were killed by cervical dislocation while under deep anesthesia.

### 4.6. PET Data Analysis

PET data were sorted into 25 time frames which duration increased from 5 s to 20 min to a total scan length of 90 min. PET images were reconstructed using Fourier re-binning of the three-dimensional sinograms followed by a two-dimensional filtered back-projection with a ramp filter giving a voxel size of 0.4 × 0.4 × 0.796 mm^3^. Using the medical image data examiner software AMIDE [31], the brain, right lung, left kidney and urinary bladder were manually outlined as regions of interest on the PET images to derive time-activity curves (TACs) expressed in units of percent of the injected dose per milliliter tissue (%ID/mL). From the log-transformed brain, lung and kidney TACs, the elimination rate constant of radioactivity washout (*k*_elim_, h^−1^) was determined by linear regression analysis from 17.5 to 80 min for the brain and from 35 to 80 min for the lung and kidney [5,6].

### 4.7. Statistical Analysis

Differences between groups were analyzed with a two-way ANOVA (row factor: mouse strain, column factor: treatment) followed by a Šidák’s multiple comparisons test using Prism 7 Software (GraphPad Software, La Jolla, CA, USA). The level of statistical significance was set to a *p* value of less than 0.05. All values are given as mean ± standard deviation (SD).

## 5. Conclusions

The putative MRP1 stimulator thiethylperazine, administered orally to wild-type and APP/PS1-21 mice at a dose of 15 mg/kg once daily for 5 days, was found ineffective to increase MRP1-mediated transport of the PET tracer [^11^C]MPG in the brain and the lungs. This may either be related to a lack of an MRP1-stimulating effect of thiethylperazine in vivo or to other factors, such as substrate-dependent MRP1 stimulation, insufficient target tissue exposure to thiethylperazine or limited sensitivity of the PET tracer to measure MRP1 stimulation.

## Figures and Tables

**Table 1:** Overview of the animal groups included in the study. The animals received a baseline scan and a scan after a 5-day oral treatment period with thiethylperazine (15 mg/kg, once daily).

Group	Scan	Weight (g)	Injected Activity (MBq)	Sex ^1^	n
Wild-type	Baseline	23.4 ± 1.4	36.3 ± 5.3	F	7
	Thiethylperazine	22.9 ± 0.6	31.4 ± 2.8		5 ^2^
APP/PS1-21	Baseline	23.5 ± 1.5	36.9 ± 3.0	F	5
	Thiethylperazine	23.9 ± 0.9	37.3 ± 7.2		
*Abcc1^(−/−)^*	Baseline	29.4 ± 4.4	34.2 ± 3.0	M/F	4/2
	Thiethylperazine	28.3 ± 5.1	41.5 ± 6.8		

^1^ M: male, F: female. ^2^ Two animals died shortly after the start of the second PET scan (at 2 and 10 min after injection of [^11^C]BMP).

**Table 2:** Elimination rate constants (*k*_elim_, h^−1^) in the brain, the right lung and the left kidney of the three mouse strains before and after treatment with thiethylperazine.

Group	Scan	Brain (h^−1^)	Lung (h^−1^)	Kidney (h^−1^)
Wild-type	Baseline	1.39 ± 0.05	1.26 ± 0.29	1.54 ± 0.31
	Thiethylperazine	1.36 ± 0.05	1.13 ± 0.24	1.72 ± 0.28
APP/PS1-21	Baseline	1.44 ± 0.11	1.26 ± 0.18	1.56 ± 0.43
	Thiethylperazine	1.43 ± 0.19	1.23 ± 0.22	1.54 ± 0.28
*Abcc1^(−/−)^*	Baseline	0.14 ± 0.01	0.30 ± 0.09	0.40 ± 0.45
	Thiethylperazine	0.18 ± 0.04	0.39 ± 0.23	0.61 ± 0.30

All data are given as mean ± SD.

**Table 3:** Radioactivity concentrations (%ID/mL) in blood and plasma and plasma-blood radioactivity concentration ratios in the three mouse strains before and after treatment with thiethylperazine.

Group	Scan	Blood (%ID/mL)	Plasma (%ID/mL)	Plasma-Blood
Wild-type	Baseline	0.35 ± 0.13	0.50 ± 0.09	1.52 ± 0.39
	Thiethylperazine	0.32 ± 0.01	0.47 ± 0.02	1.48 ± 0.04
APP/PS1-21	Baseline	0.25 ± 0.03	0.39 ± 0.06	1.53 ± 0.13
	Thiethylperazine	0.27 ± 0.10	0.41 ± 0.13	1.53 ± 0.12
*Abcc1^(−/−)^*	Baseline	0.96 ± 0.21	0.59 ± 0.09	0.63 ± 0.05
	Thiethylperazine	1.01 ± 0.28	0.58 ± 0.17	0.57 ± 0.08

Samples were obtained at the end of each PET scan (i.e., at approximately 90 min after radiotracer injection). All data are given as mean ± SD.

## Data Availability

Data is contained within the article.
